# Supplementary material for: Binding Zinc and Oxo-Vanadium Insulin-Mimetic Complexes to Phosphatase Enzymes: Structure, Electronics and Implications
Source: Molecules. 2025 Mar 26;30(7):1469. doi: 10.3390/molecules30071469 (PMC11990500; doi:10.3390/molecules30071469)
Supplement: Supplementary file 1 [file molecules-30-01469-s001.zip › SUPPLEMENTARY_MATERIAL/supplementary_material.pdf]

Supplementary Material to
"Binding Zinc and Oxo-Vanadium Insulin-Mimetic Complexes to Phosphatase Enzymes: Structure, Electronics and Implications"

Victor V. Volkov,[1,] Carole C. Perry,[2] Riccardo Chelli[3,*]*

[1] Independent Researcher, Bereozovaya 2a, Konstantinovo, Moscow Region, 140207, Russia
[2] Interdisciplinary Biomedical Research Centre, School of Science and Technology, Nottingham Trent University, Clifton Lane, Nottingham NG11 8NS, United Kingdom; carole.perry@ntu.ac.uk
[3] Dipartimento di Chimica, Università di Firenze, Via della Lastruccia 3, I-50019 Sesto Fiorentino, Italy
** Correspondence: volkovskr@gmail.com (V.V.V.); riccardo.chelli@unifi.it (R.C.)*

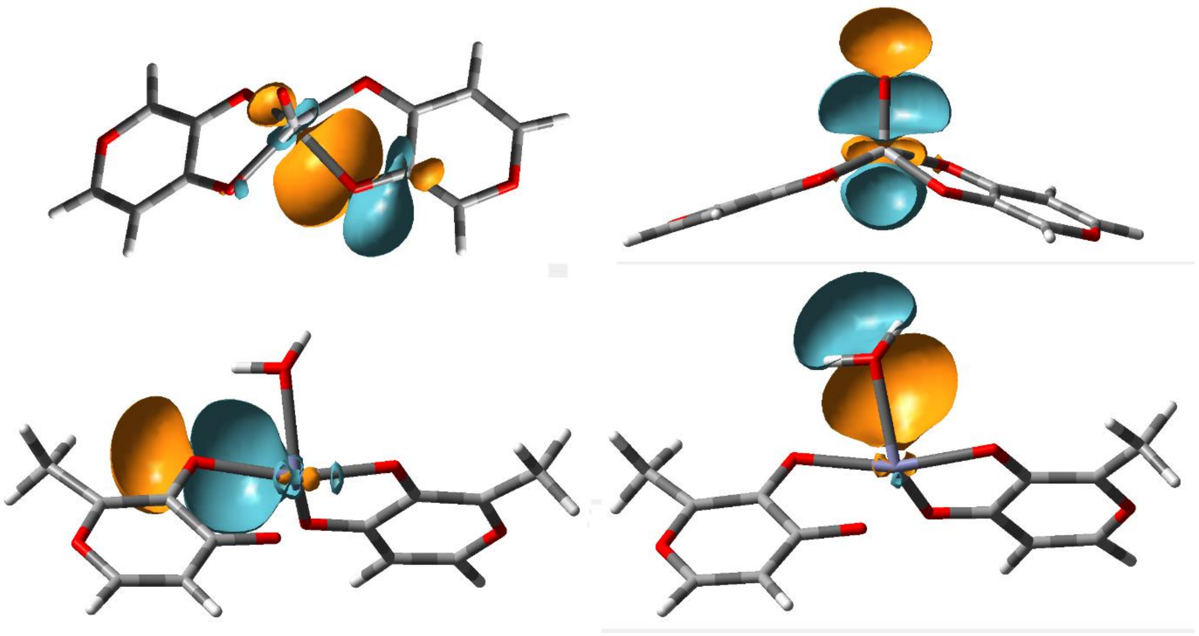

Figure S1. Nature of bonding orbital components computed for $VO(3hp)_2$ and $Zn(mal)_2 \cdot 2H_2O$

Table S1. *Properties of VO(hp)$_2$ complex using NBO analysis: : here, carbonyl and carboxylate are noted as crbn and crbx, respectively.*

bond	Length, Å	ESP charges	oxygen contribution	metal contribution
hp: V-O$_{crbn}$	2.05	-0.5985, -0.5997	88.27% s(23.53%) p$^{3.25}$(76.42%)	11.73% s(19.76%) p$^{0.96}$(18.91%) d$^{3.10}$(61.33%)
hp: V-O$_{crbx}$	1.97	-0.5478, -0.5493	87.66% s(26.00%) p$^{2.84}$(73.95%)	12.34% s(21.40%) p$^{1.39}$(29.77%) d$^{2.28}$(48.83%)
hp: V=O	1.5790	V: 1.4057	71.49% s(18.16%) p$^{4.50}$(81.74%)	28.51% s(13.17%) p$^{0.00}$(0.04%) d$^{6.59}$(86.79%)
		O: -0.5655	77.11% p$^{1.00}$(99.86%)	22.89% p$^{1.00}$(32.16%) d$^{2.11}$(67.84%)
**				
P-hp: V-O$_{crbn}$	2.04, 207	-0.5923, -0.5681	88.10% s(23.74%) p$^{3.21}$(76.21%)	11.90% s(18.66%) p$^{1.40}$(26.49%) d$^{2.79}$(54.14%)
P-hp: V-O$_{crbx}$	1.97, 1.96	-0.5183, -0.5494	86.12% s(25.27%) p$^{2.95}$(74.68%)	13.88% s(20.64%) p$^{1.25}$(25.81%) d$^{2.59}$(53.55%)
P-hp: V=O	1.57968	V: 1.292	72.92% s(21.05%) p$^{3.75}$(78.88%)	27.08% s(18.98%) p$^{0.01}$(0.23%) d$^{4.26}$(80.78%)
		O$_a$: -0.6463	77.49% p$^{1.00}$ (99.85%)	22.51% p$^{1.00}$ (30.53%) d$^{2.10}$(69.39%)

Table S2. *Properties of VO(alx)$_2$ complex using NBO analysis.*

bond	Length, Å	ESP charges	oxygen contribution	metal contribution
ax: V-O$_{crbn}$	2.047	-0.431; -0.466	88.08% s(25.25%) p$^{2.96}$(74.70%)	11.92% s(19.62%) p$^{0.89}$(17.44%) d$^{3.21}$(62.94%
ax: V-O$_{crbx}$	1.962	-0.579 ;-0.501	87.69% s(27.14%) p$^{2.68}$(72.81%)	12.31% s(21.47%) p$^{1.44}$(30.95%) d$^{2.22}$(47.58%)
ax: V=O	1.582	V: 1.323	71.64% s(18.68%) p$^{4.35}$(81.22%)	28.36% s(12.87%) p$^{0.01}$(0.10%) d$^{6.76}$(87.03%)
		O: -0.561	77.77% s(0.01%) p$^{1.00}$(99.85%)	22.23% p$^{1.00}$(33.65%) d$^{2.00}$(66.33%)
**				
P-ax: V-O$_{crbn}$	2.007, 2.074	-0.4468, -0.5440	87.09% s(24.62%) p$^{3.06}$(75.32%)	12.91% s(20.65%) p$^{1.46}$(30.20%) d$^{2.38}$(49.15%)
P-ax: V-O$_{crbx}$	2.007, 1.968	-0.5889, -0.5206	85.54% s(23.60%) p$^{3.24}$(76.34%)	14.46% s(20.61%) p$^{0.71}$(14.69%) d$^{3.14}$(64.70%)
P-ax: V=O: V	1.58402	V: 1.3356	73.78% s(21.05%) p$^{3.75}$(78.86%)	26.22% s(15.99%) p$^{0.00}$(0.05%) d$^{5.25}$(83.96%)
		O: -0.7444	79.57% s(0.22%) p$^{1.00}$(99.67%)	20.43% s(0.01%) p$^{1.00}$(31.60%) d$^{2.00}$(68.39%)

Table S3. *Properties of Zn(mal)$_2$ and Zn(mal)$_2$·H$_2$O complexes using NBO analysis.*

bond	Length, Å	ESP charges	oxygen contribution	metal contribution
mal: Zn-O$_{crbn}$	2.07	O$_{crn}$;O$_{crx}$:-0.72;-0.74	95.62% s(14.83%) p$^{5.74}$(85.10%)	4.38% s(22.66%) p$^{3.39}$(76.73%) d$^{0.03}$(0.62%)
mal: Zn-O$_{crbx}$	1.99	Zn: 1.441	94.81% s(16.71%) p$^{4.98}$(83.23%)	5.19% s(27.22%) p$^{2.65}$(72.08%) d$^{0.03}$(0.70%)
**				
mal: Zn-O7$_{crbn}$	2.10	O7;18: -0.71; -0.65	96.18% s(15.58%) p$^{5.41}$(84.36%)	3.82% s(20.82%) p$^{3.14}$(65.29%) d$^{0.67}$(13.89%)
mal: Zn-O18$_{crbn}$	2.08	O9;11: -0.57;-0.67	95.97% s(16.29%) p$^{5.13}$(83.65%)	4.03% s(22.66%) p$^{2.88}$(65.30%) d$^{0.53}$(12.04%)
mal: Zn-O9$_{crbx}$	2.03	Zn: 1.25	96.03% s(17.82%) p$^{4.61}$(82.13%)	3.97% s(23.36%) p$^{2.13}$(49.82%) d$^{1.15}$(26.81%)
mal: Zn-O11$_{crbx}$	2.08		96.67% s(16.97%) p$^{4.89}$(82.98%)	3.33% s(19.41%) p$^{2.67}$(51.81%) d$^{1.48}$(28.78%)
mal: Zn-O$_a$	2.23	Oa: -0.81	97.64% s(30.23%) p$^{2.31}$(69.72%)	2.36% s(13.77%) p$^{4.97}$(68.48%) d$^{1.29}$(17.75%)
**				
P-mal: Zn-O7$_{crbn}$	2.02121	O7;O16:-0.69;-0.82	95.62% s(14.05%) p$^{6.11}$(85.88%)	4.38% s(25.69%) p$^{2.83}$(72.61%) d$^{0.07}$(1.70%)
P-mal: Zn-O16$_{crbn}$	2.24524	O9; O23:-0.67;-0.64	97.40% s(8.40%) p$^{10.90}$(91.53%)	2.60% s(14.56%) p$^{5.72}$(83.32%) d$^{0.15}$(2.12%)
P-mal: Zn-O9$_{crbx}$	2.05197	Zn 1.34	96.16% s(12.15%) p$^{7.22}$(87.79%)	3.84% s(25.85%) p$^{2.79}$(72.08%) d$^{0.08}$(2.06%)
P-mal: Zn-O23$_{crbx}$	1.94144		95.21% s(14.87%) p$^{5.72}$(85.06%)	4.79% s(33.55%) p$^{1.92}$(64.55%) d$^{0.06}$(1.90%)

Table S4. *Properties of Zn(alx)$_2$ and Zn(alx)$_2$·H$_2$O complexes using NBO analysis.*

bond	Length, Å	ESP charges	oxygen contribution	metal contribution
ax: Zn-O$_{crbn}$	2.07	O$_{crn}$;O$_{crx}$:-0.6;-0.70	95.76% s(14.38%) p$^{5.95}$(85.56%)	4.24% s(22.11%) p$^{3.49}$(77.22%) d$^{0.03}$(0.66%)
ax: Zn-O$_{crbx}$	1.98	Zn: 1.396	94.76% s(16.67%) p$^{5.01}$(83.27%)	5.24% s(27.78%) p$^{2.57}$(71.51%) d$^{0.03}$(0.72%)
**				
ax: Zn-O7$_{crbn}$	2.08270	O7;O18:-0.53;-0.60	96.10% s(15.69%) p$^{5.37}$(84.26%)	3.90% s(22.83%) p$^{2.81}$(64.18%) d$^{0.57}$(12.99%)
ax: Zn-O18$_{crbn}$	2.10554	O9;O30: -0.70;-0.64	96.30% s(15.01%) p$^{5.66}$(84.93%)	3.70% s(20.42%) p$^{3.23}$(65.96%) d$^{0.67}$(13.62%)
ax: Zn-O9$_{crbx}$	2.08411	Zn: 1.24	96.69% s(16.99%) p$^{4.88}$(82.97%)	3.31% s(19.33%) p$^{2.68}$(51.78%) d$^{1.50}$(28.89%)
ax: Zn-O30$_{crbx}$	2.01984		96.00% s(17.68%) p$^{4.65}$(82.26%)	4.00% s(23.74%) p$^{2.10}$(49.74%) d$^{1.12}$(26.52%)
ax: Zn-O$_a$	2.24549	O: -0.788	97.68% s(29.13%) p$^{2.43}$(70.83%)	2.32% s(13.75%) p$^{5.02}$(69.08%) d$^{1.25}$(17.16%)
**				
P-ax: Zn-O7$_{crbn}$	2.08442	O7;O35: -0.52;-0.85	96.41% s(11.77%) p$^{7.49}$(88.17%)	3.59% s(20.88%) p$^{3.75}$(78.36%) d$^{0.04}$(0.76%)
P-ax: Zn-O35$_{crbn}$	2.18540	O9;O42:-0.70; -0.61	96.75% s(10.42%) p$^{8.59}$(89.53%)	3.25% s(15.75%) p$^{5.30}$(83.53%) d$^{0.05}$(0.71%)
P-ax: Zn-O9$_{crbx}$	2.02549	Zn: 1.326	95.39% s(13.77%) p$^{6.26}$(86.18%)	4.61% s(27.34%) p$^{2.63}$(71.93%) d$^{0.03}$(0.73%)
P-ax: Zn-O42$_{crbx}$	1.93790		94.05% s(15.57%) p$^{5.42}$(84.37%)	5.95% s(35.64%) p$^{1.78}$(63.54%) d$^{0.02}$(0.82%)

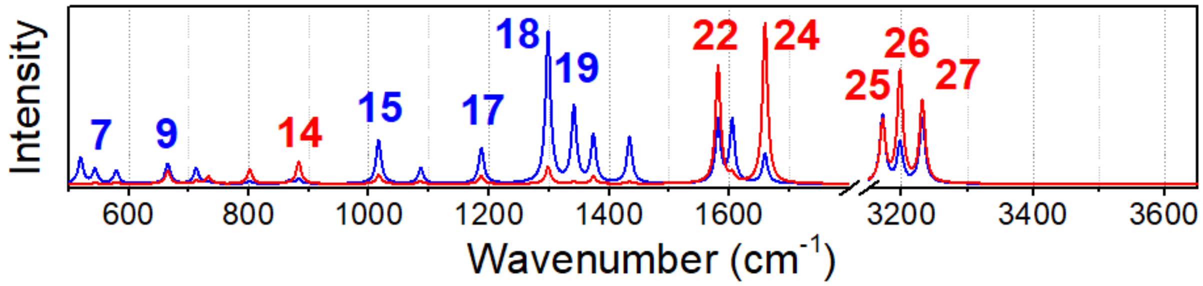

Figure S2. Infrared (red line) and Raman (blue line) spectra computed for deprotonated 3-hydroxy-4-pyrone. Intensities of spectra in the middle and high frequency ranges are scaled for comparative visibility. Raman spectrum is computed accounting excitation wavelength at 532 nm.

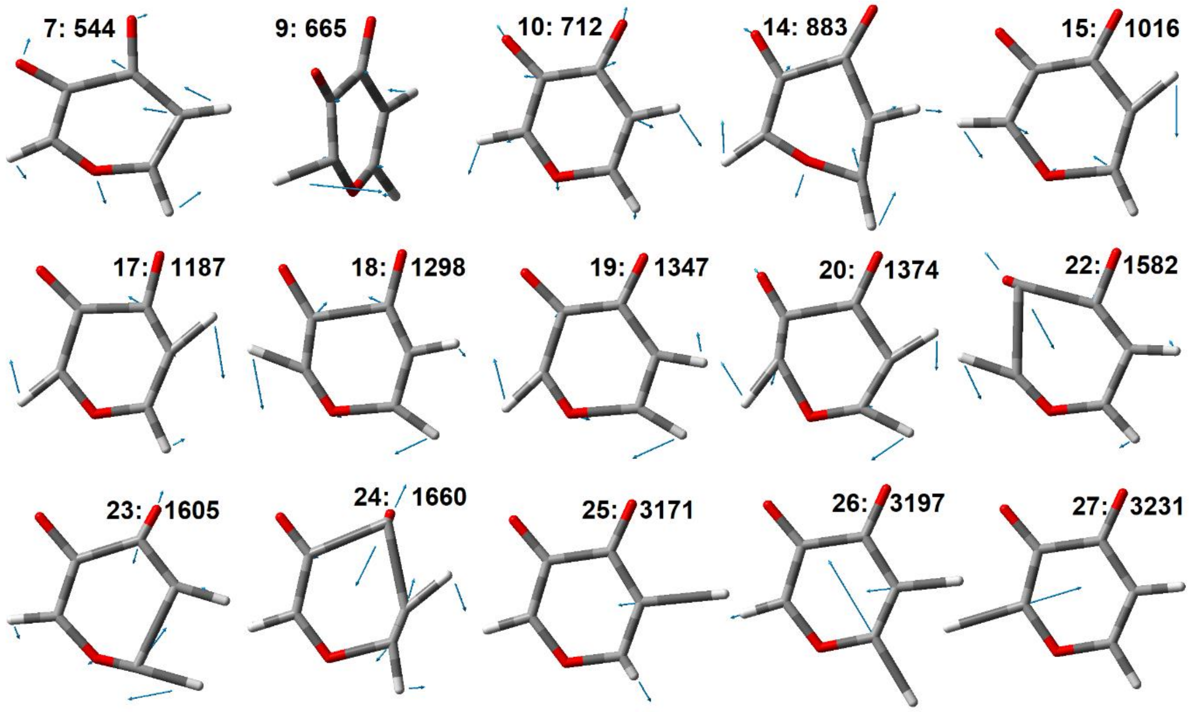

Figure S3. Images of displacements for selected normal modes as computed for deprotonated 3-hydroxy-4-pyrone. For each normal mode the numbering used in the Fig. S2 is reported together with the corresponding frequency in cm^{-1}.

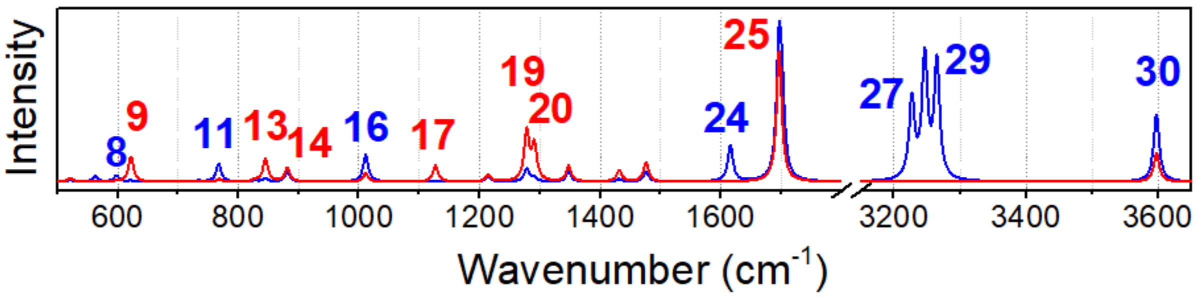

Figure S4. Infrared (red line) and Raman (blue line) spectra computed for protonated 3-hydroxy-4-pyrone. Intensities of spectra in the middle and high frequency ranges are scaled for comparative visibility. Raman spectrum is computed accounting excitation wavelength at 532 nm.

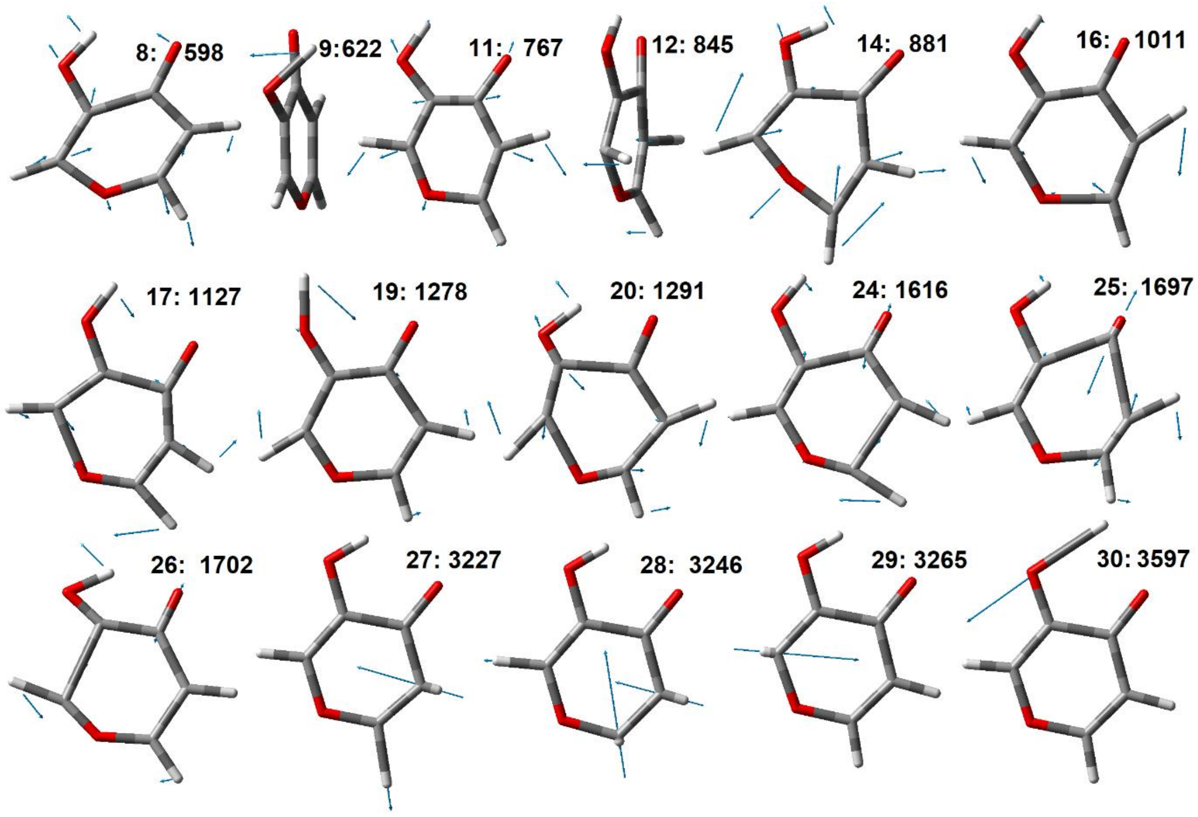

Figure S5. Images of displacements for selected normal modes as computed for protonated 3-hydroxy-4-pyrone. For each normal mode the numbering used in the Fig. S4 is reported together with the corresponding frequency in cm^{-1}.

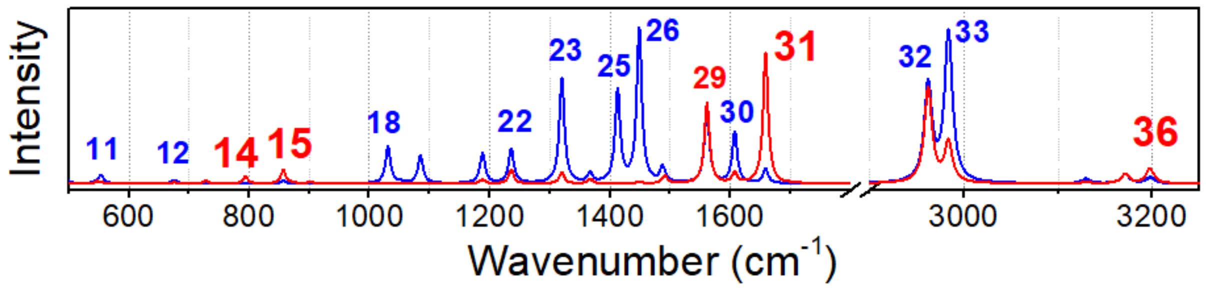

Figure S6. Infrared (red line) and Raman (blue line) spectra computed for deprotonated maltol. Intensities of spectra in the middle and high frequency ranges are scaled for comparative visibility. Raman spectrum is computed accounting excitation wavelength at 532 nm. The results present a detailed perspective considering previous experimental and theoretical studies for maltol anion [58].

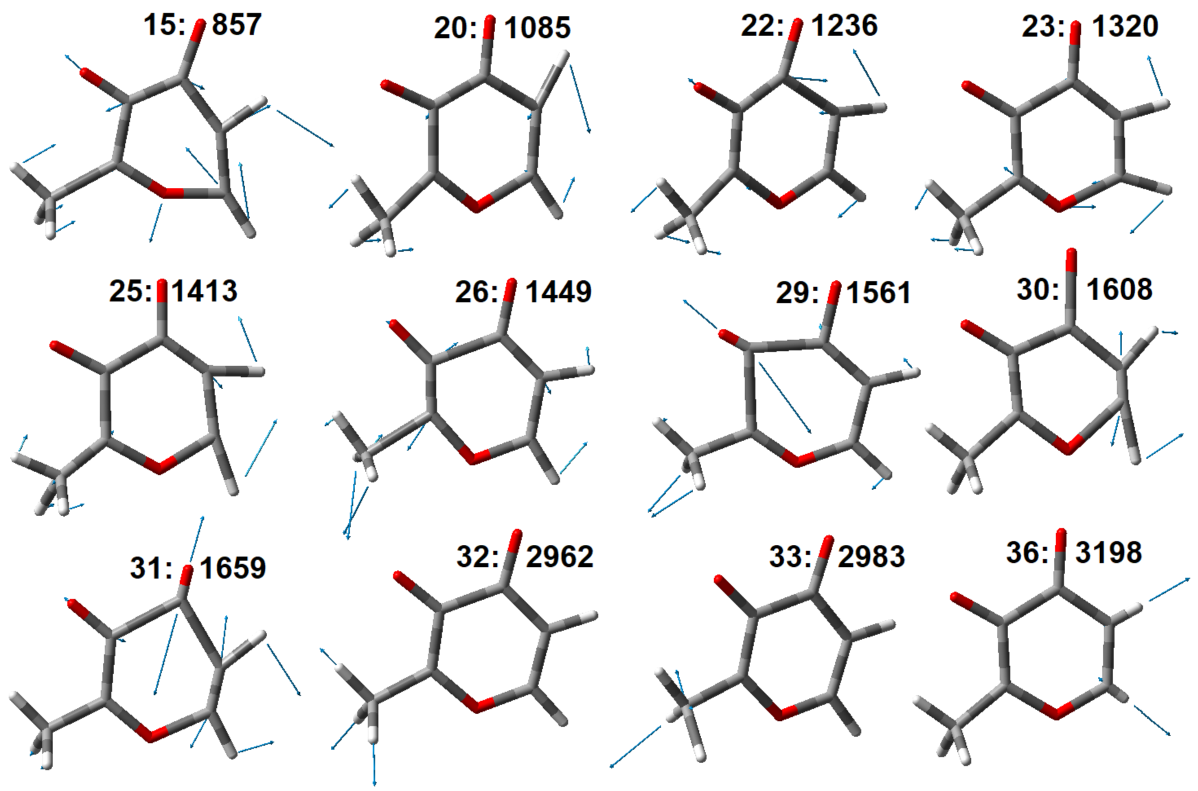

Figure S7. Images of displacements for selected normal modes as computed for deprotonated maltol. The results present a detailed perspective considering previous experimental and theoretical studies for maltol anion [58]. For each normal mode the numbering used in the Fig. S6 is reported together with the corresponding frequency in cm^{-1}.

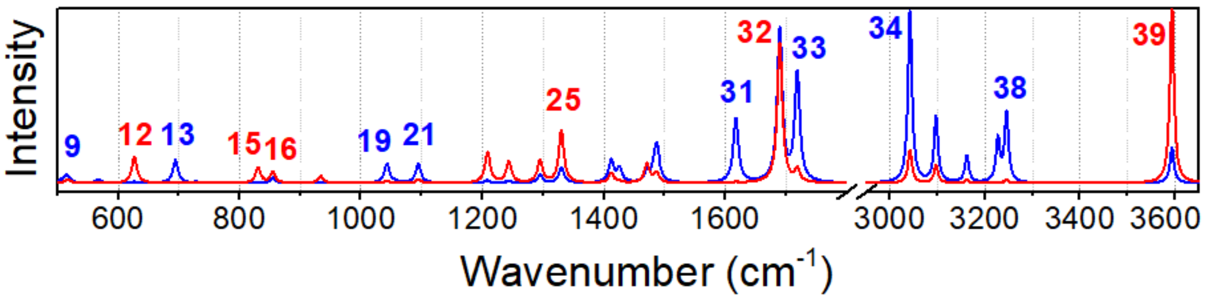

Figure S8. Infrared (red line) and Raman (blue line) spectra computed for protonated maltol. Intensities of spectra in the middle and high frequency ranges are scaled for comparative visibility. Raman spectrum is computed accounting excitation wavelength at 532 nm. The results present a detailed perspective considering previous experimental and theoretical studies for neutral maltol [58].

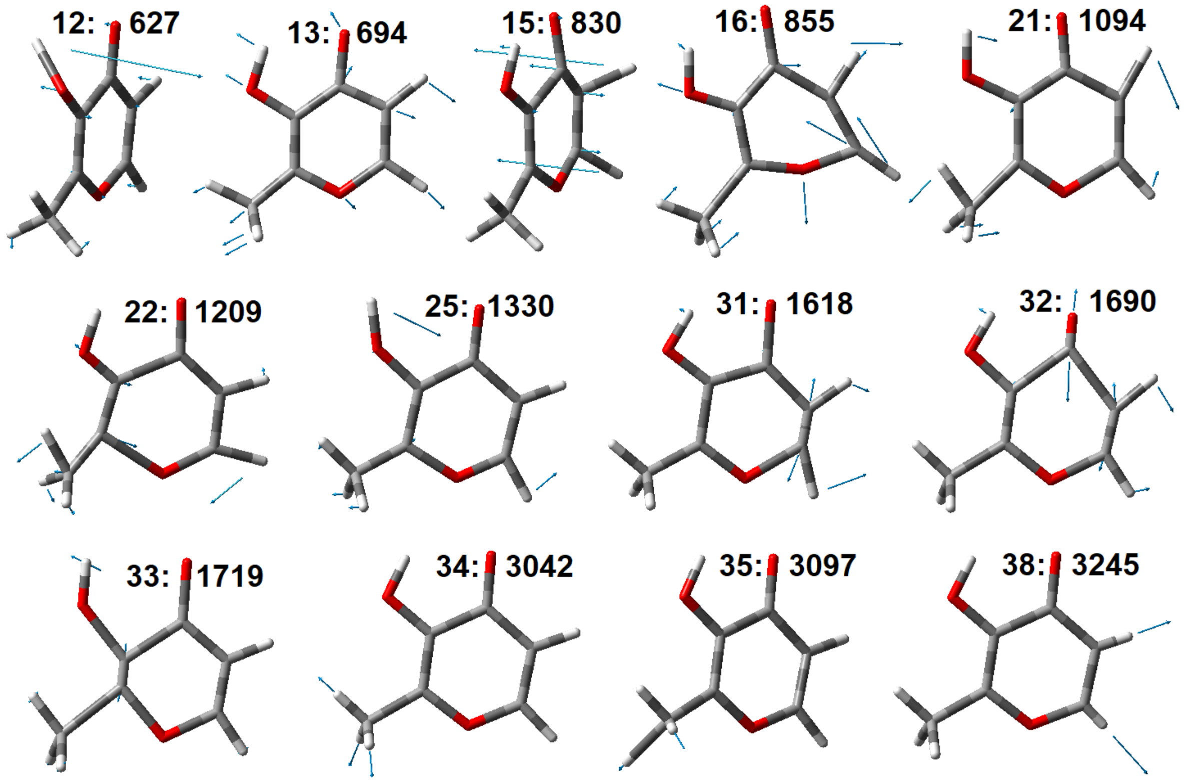

Figure S9. Images of displacements for selected normal modes as computed for protonated maltol. The results present a detailed perspective considering previous experimental and theoretical studies for neutral maltol [58]. For each normal mode the numbering used in the Fig. S2 is reported together with the corresponding frequency in cm^{-1}.

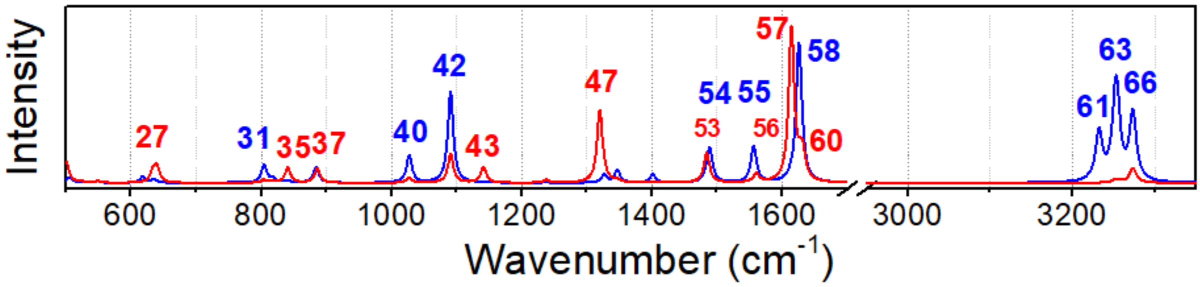

Figure S10. Infrared (red line) and Raman (blue line) spectra computed for $VO(3hp)_2$. Intensities of spectra in the middle and high frequency ranges are scaled for comparative visibility. Raman spectrum is computed accounting excitation wavelength at 532 nm.

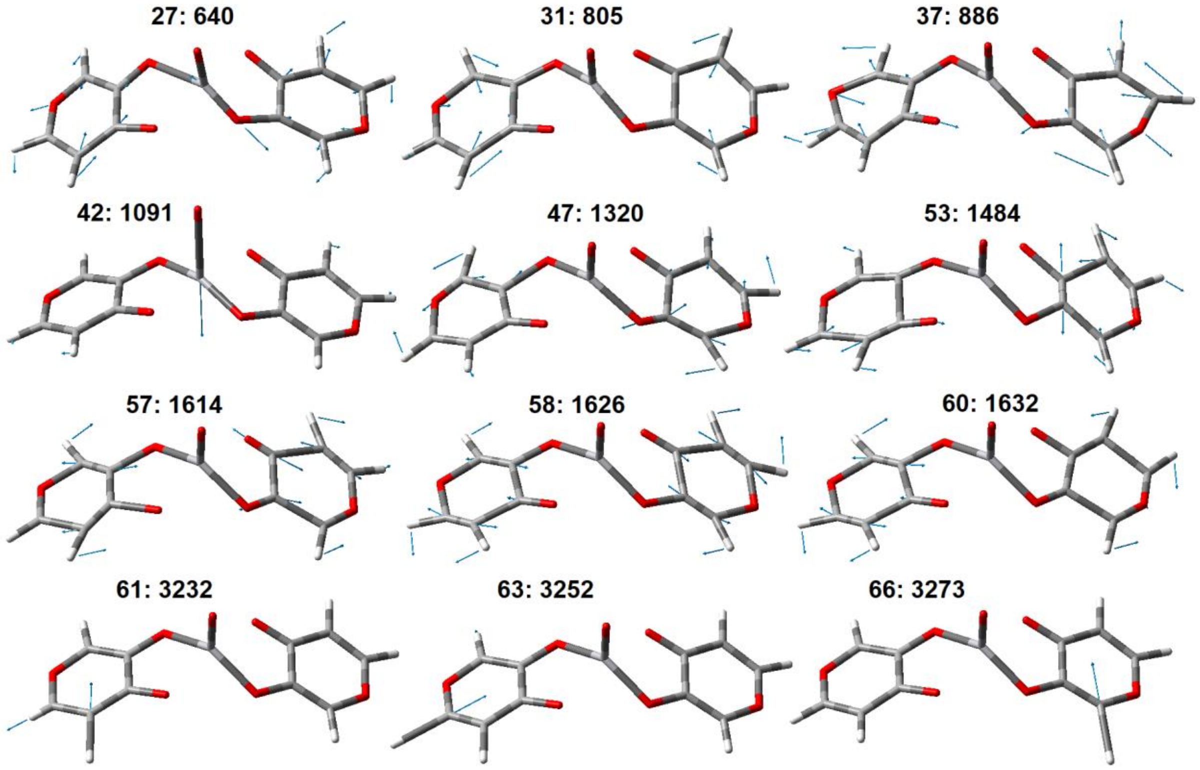

Figure S11. Images of displacements for selected normal modes as computed for $VO(3hp)_2$. For each normal mode the numbering used in the Fig. S10 is reported together with the corresponding frequency in cm^{-1}.

In Figure S10, we compare IR and Raman spectra of the considered complexes in vacuum. The normal mode 57 (at 1614 cm^{-1}) dominates the IR spectrum. This is a stretching mode localized on both ligands. In particular, it presents a C_3-O_3&C_3-O_4 symmetric stretching accompanied by an out-of-phase C_2-C_3/C_4-C_5 symmetric stretching. We use the ampersand and the slash to indicate in-phase and out-of-phase motion, respectively. Such a motion occurs in opposite phase on the two ligands. Normal mode 58 (at 1626 cm^{-1}) dominates the Raman spectrum. This vibration accounts for a C_2-C_3&C_5-C_6 symmetric stretching in phase on both ligands. At lower frequencies, aromatic CH in-plane bending modes 40, 41 and V=O stretching mode 42 present distinct resonances, which can be helpful to identify this molecule experimentally. Aromatic CH stretching modes dominate the spectra at the highest frequencies (above 3200 cm^{-1}).

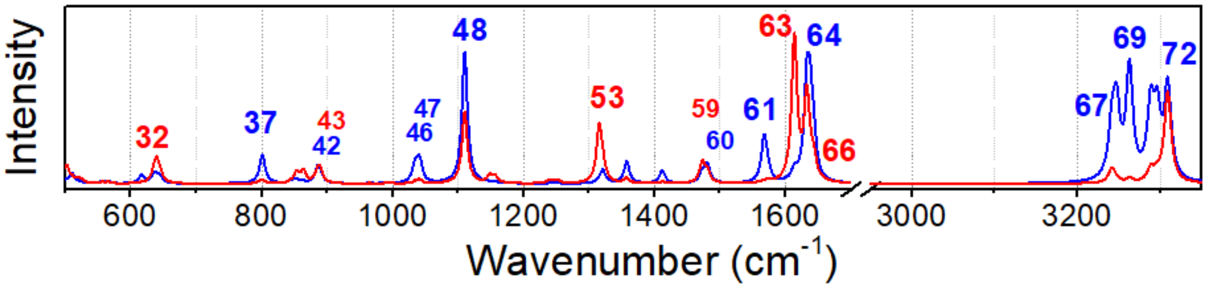

Figure S12. Infrared (red line) and Raman (blue line) spectra computed for $VO(3hp)_2$, when associated with PTP1B. Modes' numbering account additional 3 translations and 3 rotations of the complex in respect to protein cavity: they are the lowest frequency modes. Intensities of spectra in the middle and high frequency ranges are scaled for comparative visibility. Raman spectrum is computed accounting excitation wavelength at 532 nm.

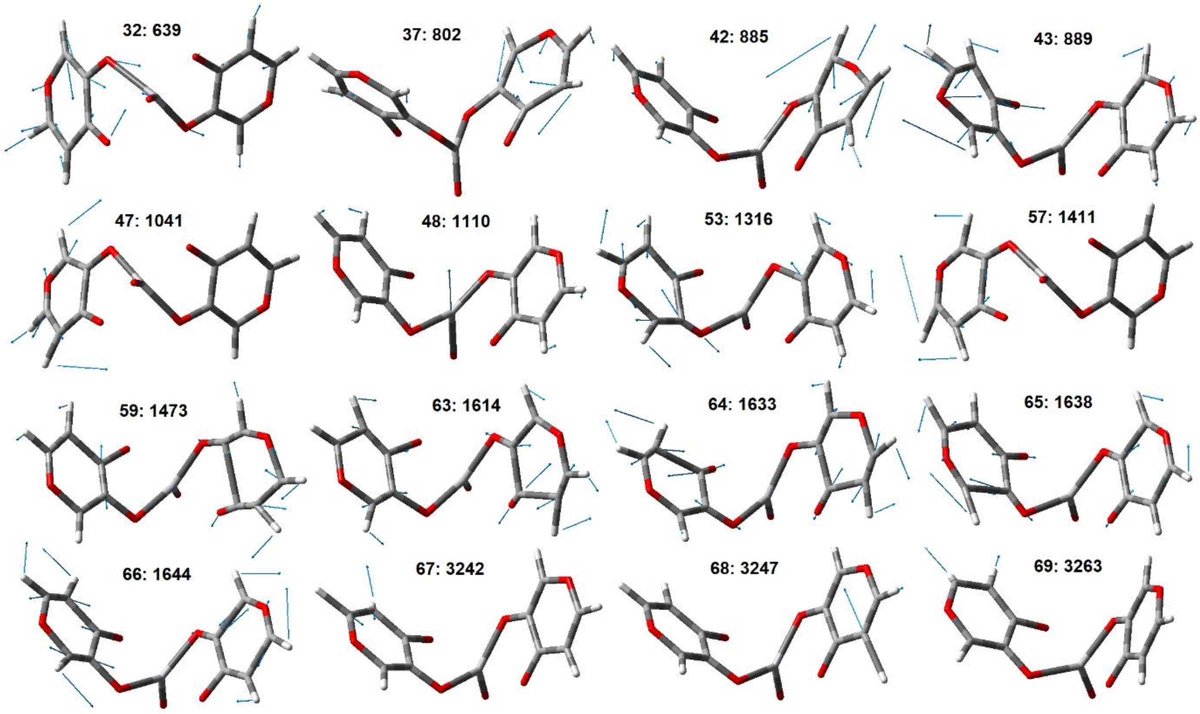

Figure S13. Images of displacements for selected normal modes as computed for $VO(3hp)_2$, when associated with PTP1B. For each normal mode the numbering used in the Fig. S12 is reported together with the corresponding frequency in cm^{-1}.

In the protein environment, 6 additional low frequency modes occur, namely, 3 translations and 3 rotations of the whole complex in respect to the protein cavity. Consequently, for example, mode 48 = 42 + 6 in Figure S12 presents V=O stretching of the complex, when in the protein environment. Comparing the spectra of Figures S10 and S12, we note that, in general, normal modes experience blue shifts upon association with the protein. Furthermore, a change of relative IR intensities of the modes 63 and 64 (versus the corresponding modes 57 and 58), as well as degeneracy lifting at 3246 cm^{-1} in Figure S12 suggest a loss of symmetry in the complex due to distortion upon association with the protein (see description of the structural properties in the previous section).

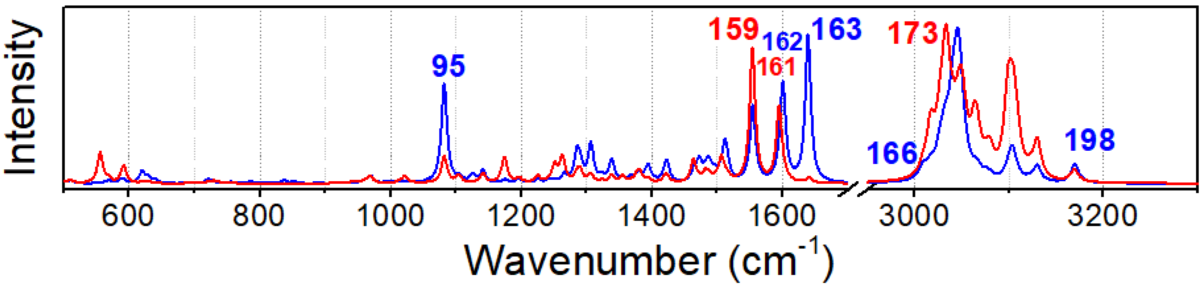

Figure S14. Infrared (red line) and Raman (blue line) spectra computed for VO(alx)$_2$. Intensities of spectra in the middle and high frequency ranges are scaled for comparative visibility. Raman spectrum is computed accounting excitation wavelength at 532 nm.

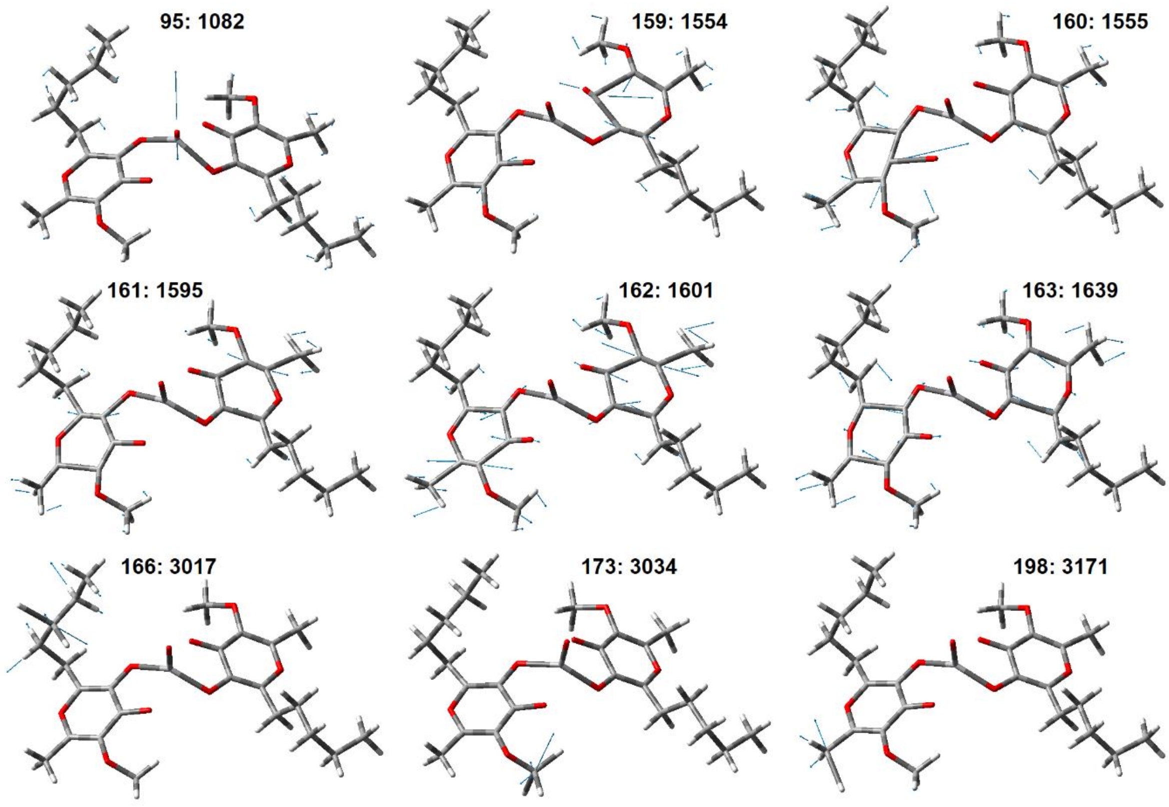

Figure S15. Images of displacements for selected normal modes as computed for VO(alx)$_2$. For each normal mode the numbering used in the Fig. S14 is reported together with the corresponding frequency in cm^{-1}.

The Raman intense mode 95, at 1082 cm^{-1} (Figure S14), is due to V=O stretching. Given its dominant role in the spectral region around 1100 cm^{-1}, this mode may serve as a helpful spectral marker to track the presence of such a complex. The same role could be played by the intense IR active modes 159 and 160 (at 1554 and 1544 cm^{-1}), which are C=O antisymmetric and symmetric stretchings, respectively. Consistently, the intense IR mode 161 (at 1595 cm^{-1}) is a delocalized stretching to involve both ligands as (C_2-C_3/C_5-C_6)&(C'_2-C'_3/C'_5-C'_6). Considering Raman diagnostics, theory predicts efficient scattering for the mode 162 (at 1601 cm^{-1}), which accounts for (C_2-C_3/C_5-C_6)/(C'_2-C'_3/C'_5-C'_6) stretching; and the mode 163 (at 1639 cm^{-1}), which is due to in phase (C_2-C_3&C_5-C_6)&(C'_2-C'_3&C'_5-C'_6) symmetric stretchings that involves the two ligands. In the high frequency range, CH_2 and CH_3 stretching modes of the side groups define a series of transitions between 2900 and 3200 cm^{-1}. If *in vitro*, such transitions occur in the same spectral region of phospholipids and proteins, probably yielding band overlap thus making them ineffective as spectral markers.

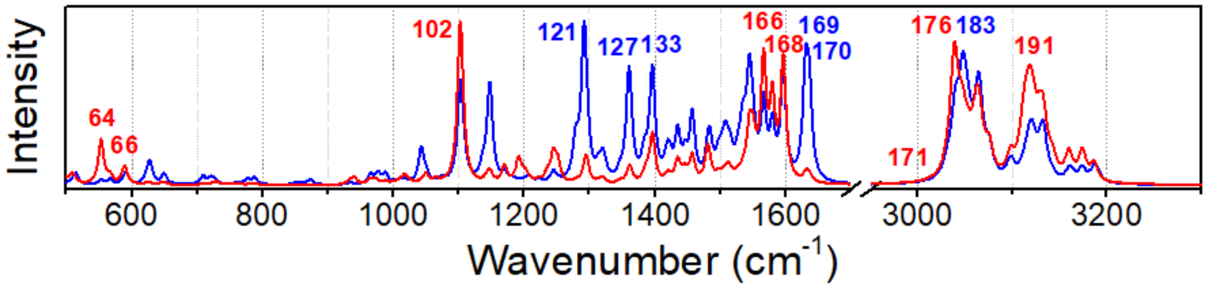

Figure S16. Infrared (red line) and Raman (blue line) spectra computed for $VO(alx)_2$, when associated with PTP1B. Modes' numbering account additional 3 translations and 3 rotations of the complex in respect to protein cavity: they are the lowest frequency modes. Intensities of spectra in the middle and high frequency ranges are scaled for comparative visibility. Raman spectrum is computed accounting excitation wavelength at 532 nm.

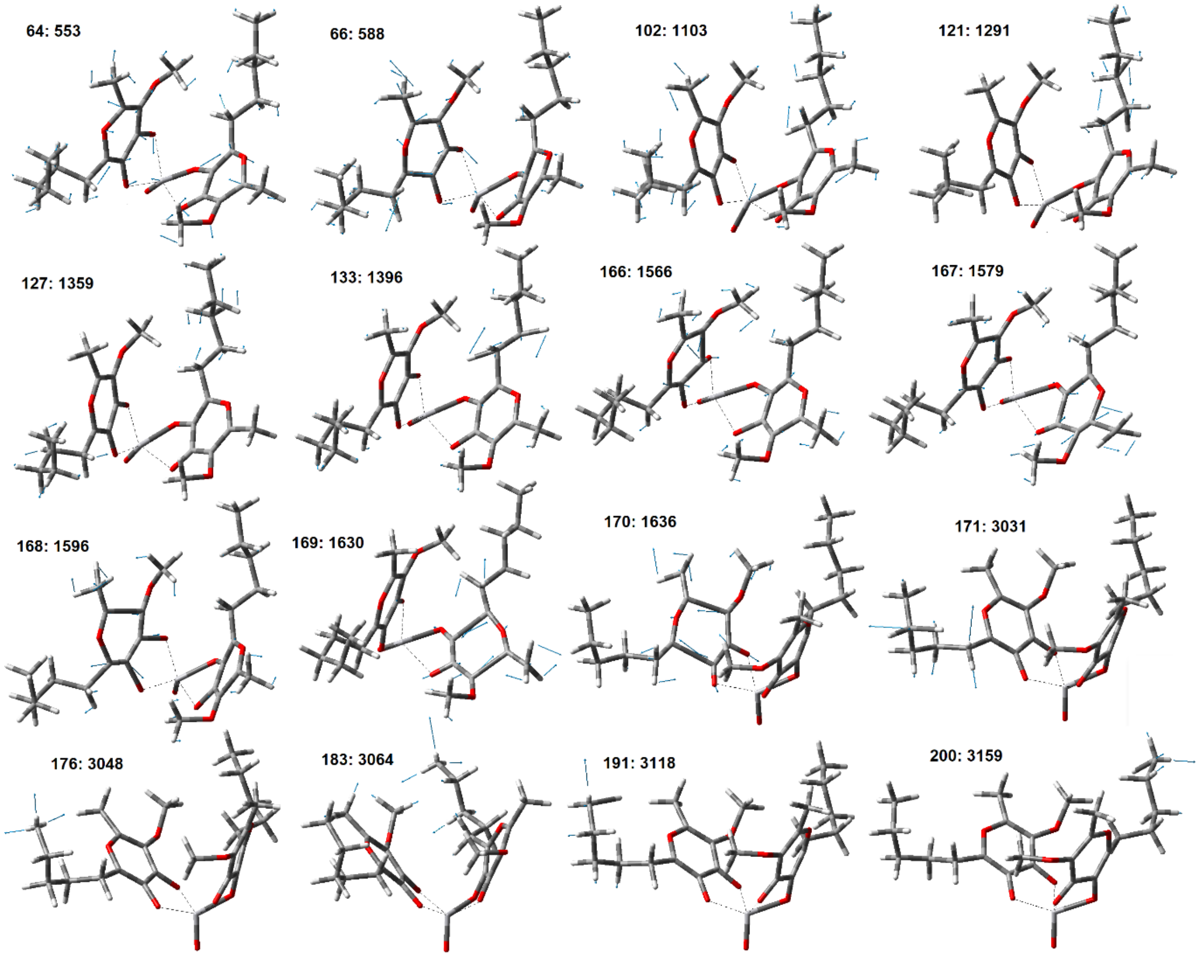

Figure S17. Images of displacements for selected normal modes as computed for $VO(alx)_2$, when associated with PTP1B. For each normal mode the numbering used in the Fig. S16 is reported together with the corresponding frequency in cm^{-1}.

In the protein environment, 6 additional low frequency modes are expected for $VO(alx)_2$: 3 translations and 3 rotations of the whole complex in respect to the protein cavity. Consequently, for $VO(alx)_2$ associated with PTP1B (see Figure S16), normal mode 95 + 6 = 101 (at 1101 cm^{-1}) is specific to V=O stretching, but not uniquely if we compare with the case of $VO(3hp)_2$.

Specifically, in the case of $VO(alx)_2$ in PTP1B, theory computes mixing of V=O stretching with delocalized CH_2 bendings: there is another intense transition denoted as 102 (1103 cm^{-1}). Further, for the complex in the protein environment, there are two intense IR modes 166 (at 1566 cm^{-1}) and 168 (at 1596 cm^{-1}), as well as a strong Raman mode 170 (at 1636 cm^{-1}) localized on the same ligand.

Mode 166 is due to (C_4-O_4&C_2-C_3)/C_4-C_5 stretching: here, the slash indicates opposite phase relations.

Mode 168 accounts (C_5-C_6&C_3-O_3&C_4-O_4)/C_2-C_3 stretching admixed with CH_3 bendings. Mode 170 represents (C_2-C_3&C_5-C_6)/C_4-O_4 stretching admixed with CH_2 and CH_3 bendings. In the considered spectral region, the other ligand demonstrates only one intense Raman mode 169 (at 1630 cm^{-1}) to account (C'_2-C'_3&C'_5-C'_6)/C'_4-O'_4 stretching admixed with CH_2 and CH_3 bendings.

The results of our theoretical studies suggest great sensitivity of the complex vibrational properties to anisotropy of interactions within the embedding cavity.

We believe the provided description of the nature of the most intense normal modes may allow a helpful diagnostic to address the binding process if using FTIR and Raman spectroscopy

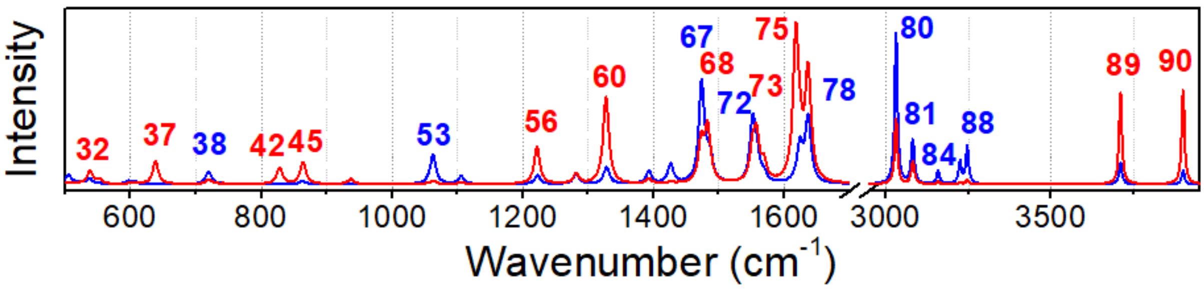

Figure S18. Infrared (red line) and Raman (blue line) spectra computed for Zn(mal)$_2$·H_2O. Intensities of spectra in the middle and high frequency ranges are scaled for comparative visibility. Raman spectrum is computed accounting excitation wavelength at 532 nm. Considering experimental results reported previously [59], we may ascribe infrared transitions observed at 852, 922, 1202, 1277 1459, 1513, 1577 and 1610 cm^{-1} to the computed modes 42, 45, 56, 60, 68, 73, 75 and 77+78, respectively. Consistently, we may ascribe Raman transitions detected at 539, 718, 1045, 1366, 1465, 1511 and 1602 cm^{-1} to modes 31, 38+39, 53, 62, 67, 72, and 78, respectively.

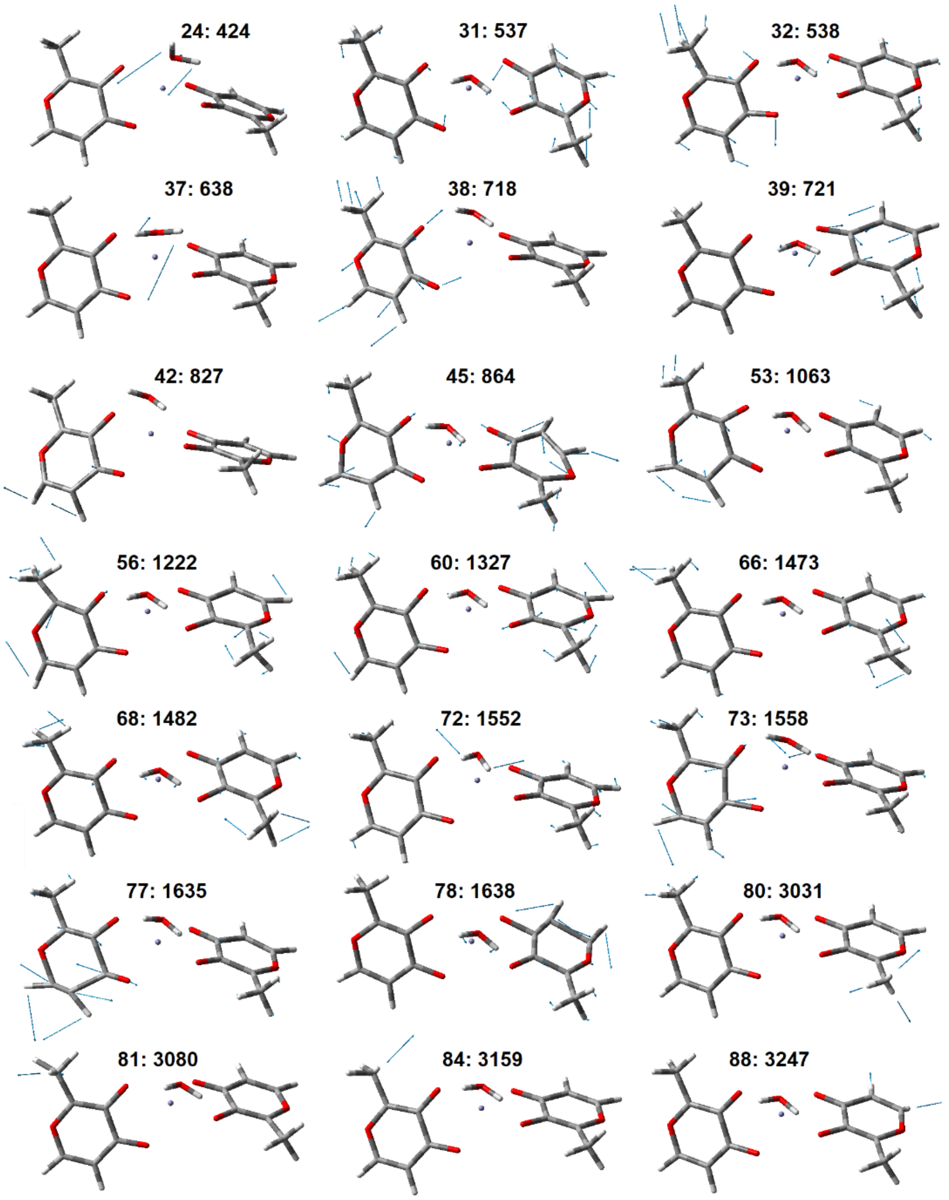

Figure S19. Images of displacements for selected normal modes as computed for Zn(mal)$_2$·H_2O. For each normal mode the numbering used in the Fig. S18 is reported together with the corresponding frequency in cm^{-1}.

Concerning the $Zn(mal)_2{\cdot}H_2O$ complex in vacuum, theory predicts IR active modes 56 and 60 to arise from aromatic CH in-plane bending admixed with O_1-C_2 stretching, with methyl CH in-plane bending and of carbonyl and carboxylate stretching on both ligands, under different phase relations. Mode 75 is an analogue of mode 57 in $VO(3hp)_2$. IR activities 77 and 78 are related to motions localized on each of the two ligands, accounting for in phase C_5-C_6&C_4-O_4 symmetric stretching admixed with aromatic C-H in-plane bendings. The two localizations are due to the low symmetry of $Zn(mal)_2{\cdot}H_2O$. In the high frequency spectral range, aromatic CH stretchings at 3150 cm^{-1} accompany CH stretchings of the methyl group within the 2900 - 3000 cm^{-1} spectral range.

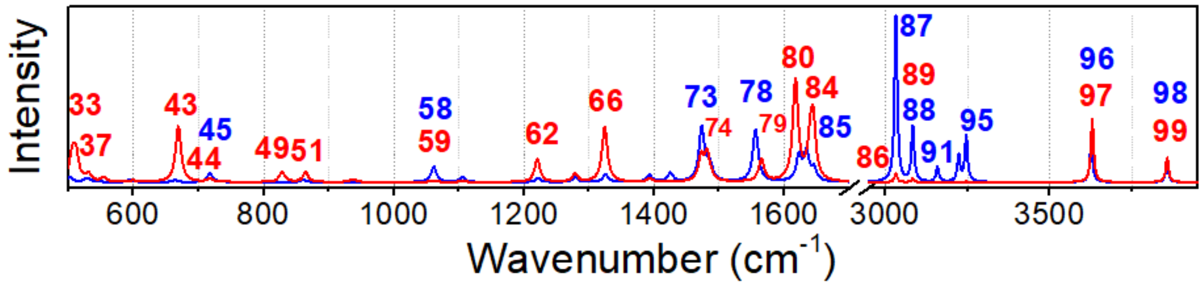

Figure S20. Infrared (red line) and Raman (blue line) spectra computed for $Zn(mal)_2{\cdot}2H_2O$ under octahedral geometry. Intensities of spectra in the middle and high frequency ranges are scaled for comparative visibility. Raman spectrum is computed accounting excitation wavelength at 532 nm.

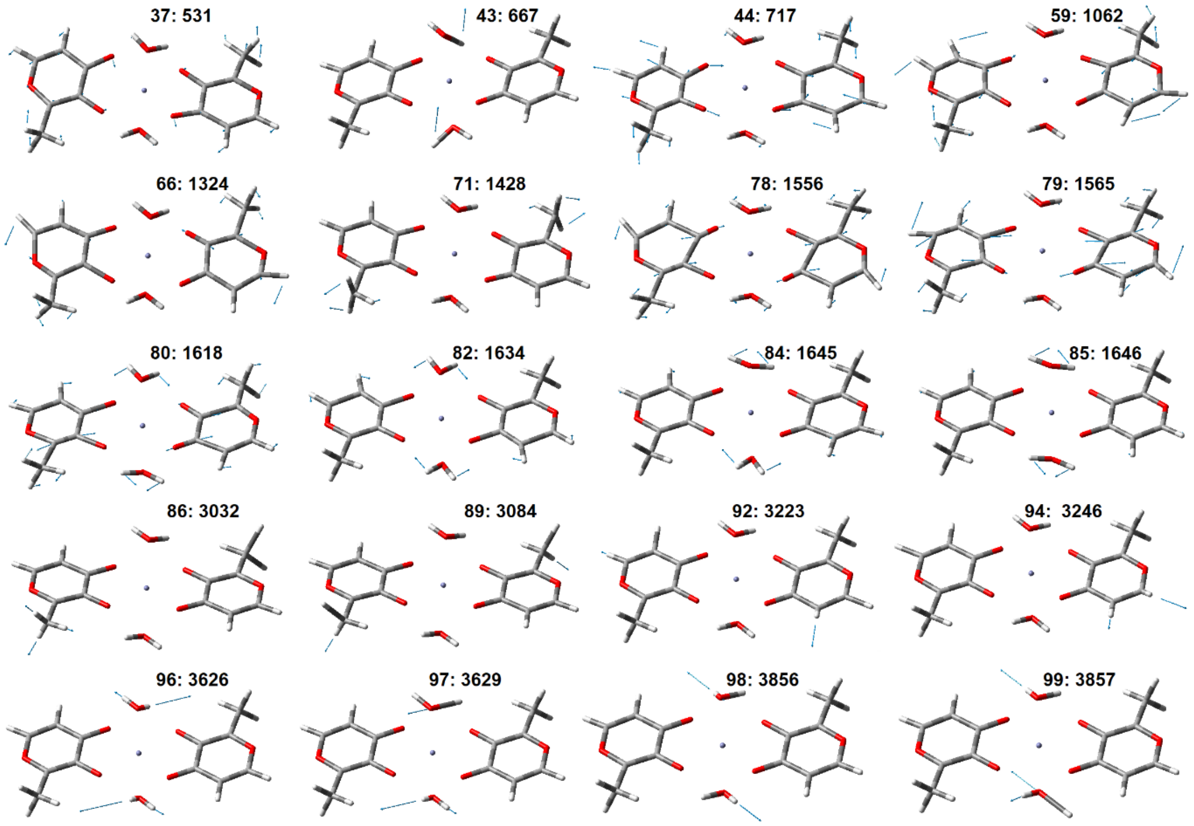

Figure S21. Images of displacements for selected normal modes as computed for $Zn(mal)_2{\cdot}2H_2O$ under octahedral geometry. For each normal mode the numbering used in the Fig. S20 is reported together with the corresponding frequency in cm^{-1}.

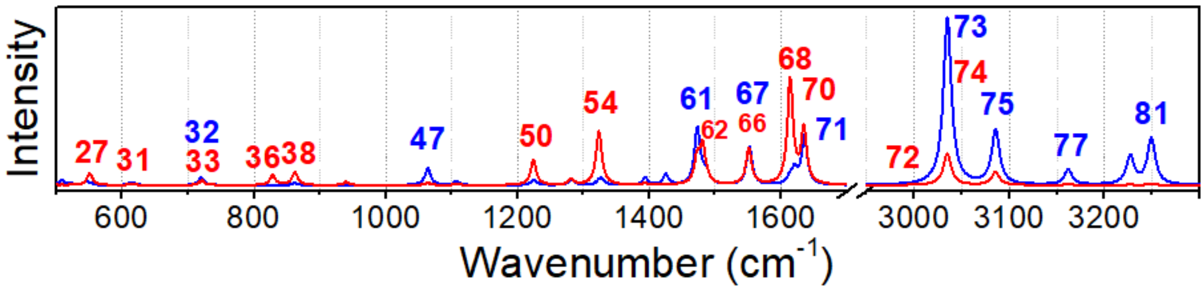

Figure S22. Infrared (red line) and Raman (blue line) spectra computed for Zn(mal)$_2$ under tetrahedral geometry. Intensities of spectra in the middle and high frequency ranges are scaled for comparative visibility. Raman spectrum is computed accounting excitation wavelength at 532 nm.

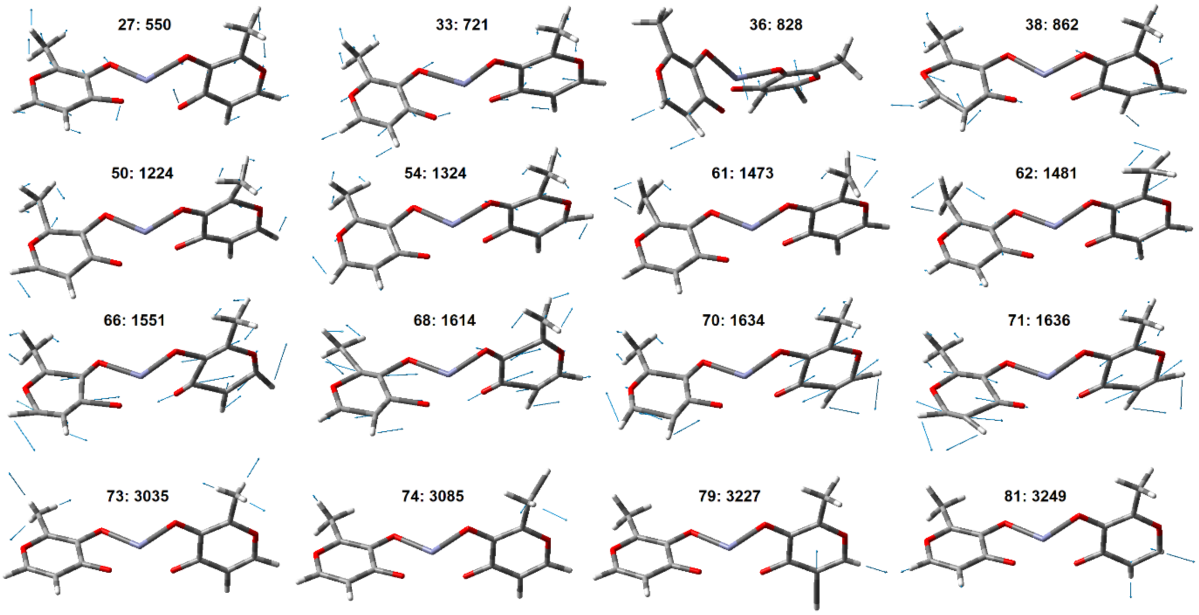

Figure S23. Images of displacements for selected normal modes as computed for Zn(mal)$_2$ under tetrahedral geometry. For each normal mode the numbering used in the Fig. S22 is reported together with the corresponding frequency in cm^{-1}.

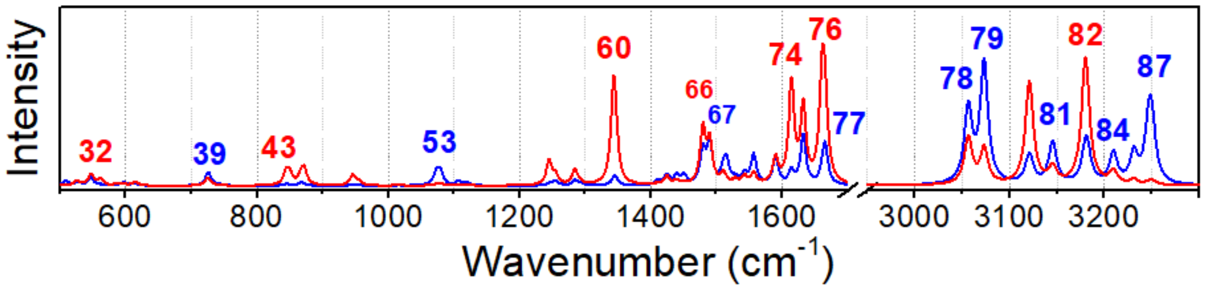

Figure S24. Infrared (red line) and Raman (blue line) spectra computed for Zn(mal)$_2$, when associated with PTEN. Modes' numbering account additional 3 translations and 3 rotations of the complex in respect to protein cavity: they are the lowest frequency modes. Intensities of spectra in the middle and high frequency ranges are scaled for comparative visibility. Raman spectrum is computed accounting excitation wavelength at 532 nm.

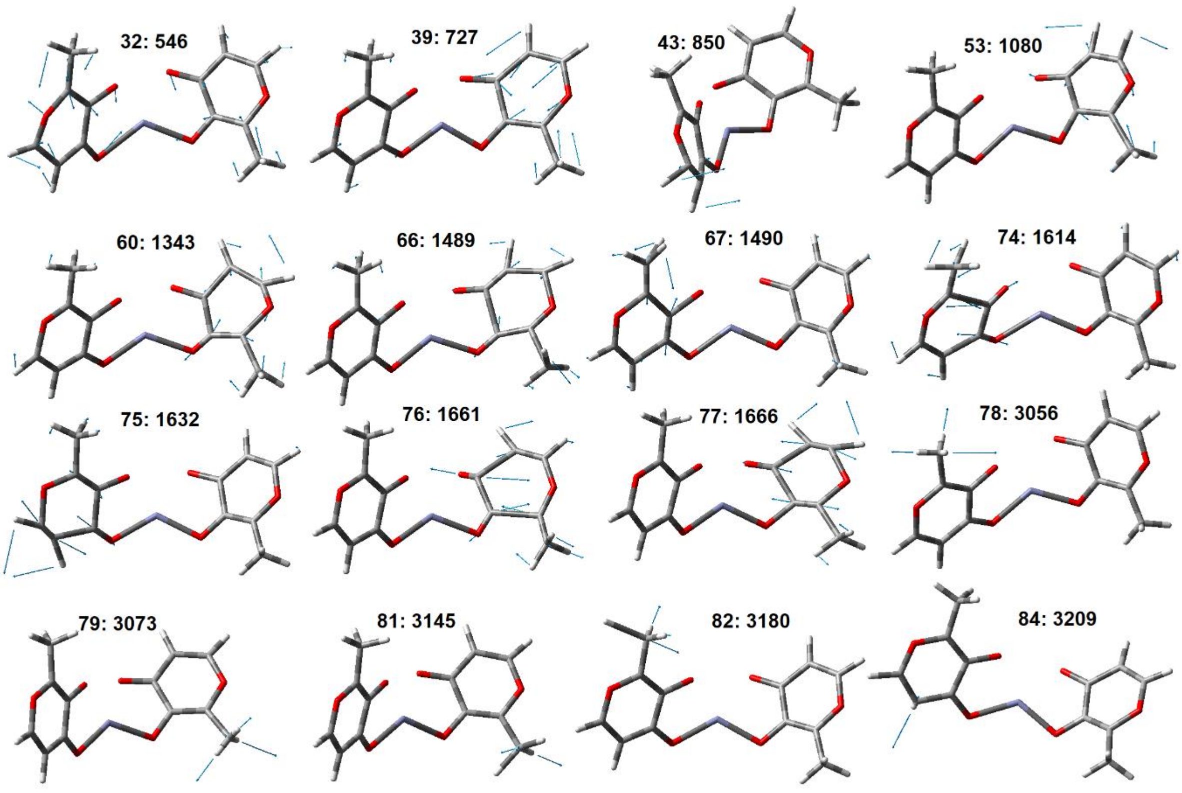

Figure S25. Images of displacements for selected normal modes as computed for Zn(mal)$_2$, when associated with PTEN. For each normal mode the numbering used in the Fig. S24 is reported together with the corresponding frequency in cm^{-1}.

One may easily note comparing Figures S18 and S25, normal modes of Zn(mal)$_2$ shift to the blue upon binding to the protein, also revealing some degree of stronger dispersion of the frequencies. These are the signatures of structural distortion and symmetry lowering. Our results indicate that, the modes 74 (at 1614 cm^{-1}) and 76 (at 1661 cm^{-1}) of Zn(mal)$_2$ in PTEN may provide valuable experimental markers of the presence and the structural state of Zn(mal)$_2$. The mode 74 accounts C_2-C_3/C_4-O_4 out-of-phase symmetric stretching admixed with CH bendings to localize on one of the ligands. The other mode accounts analogous vibration but to localize on the other ligands.

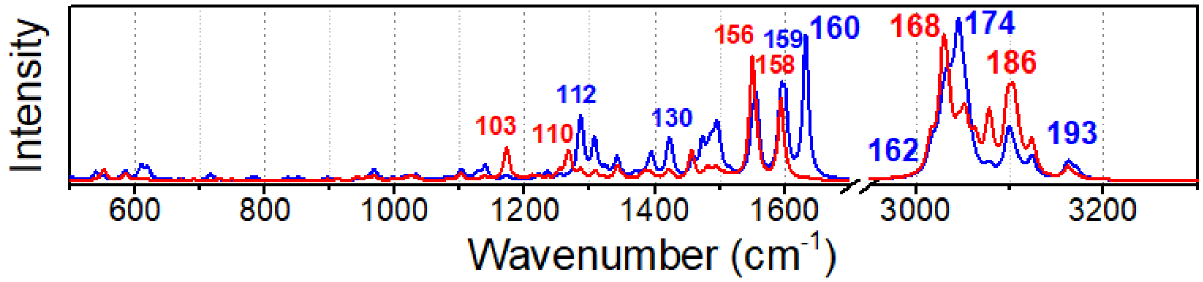

Figure S26. Infrared (red line) and Raman (blue line) spectra computed for Zn(alx)$_2$ under tetrahedral geometry. Intensities of spectra in the middle and high frequency ranges are scaled for comparative visibility. Raman spectrum is computed accounting excitation wavelength at 532 nm.

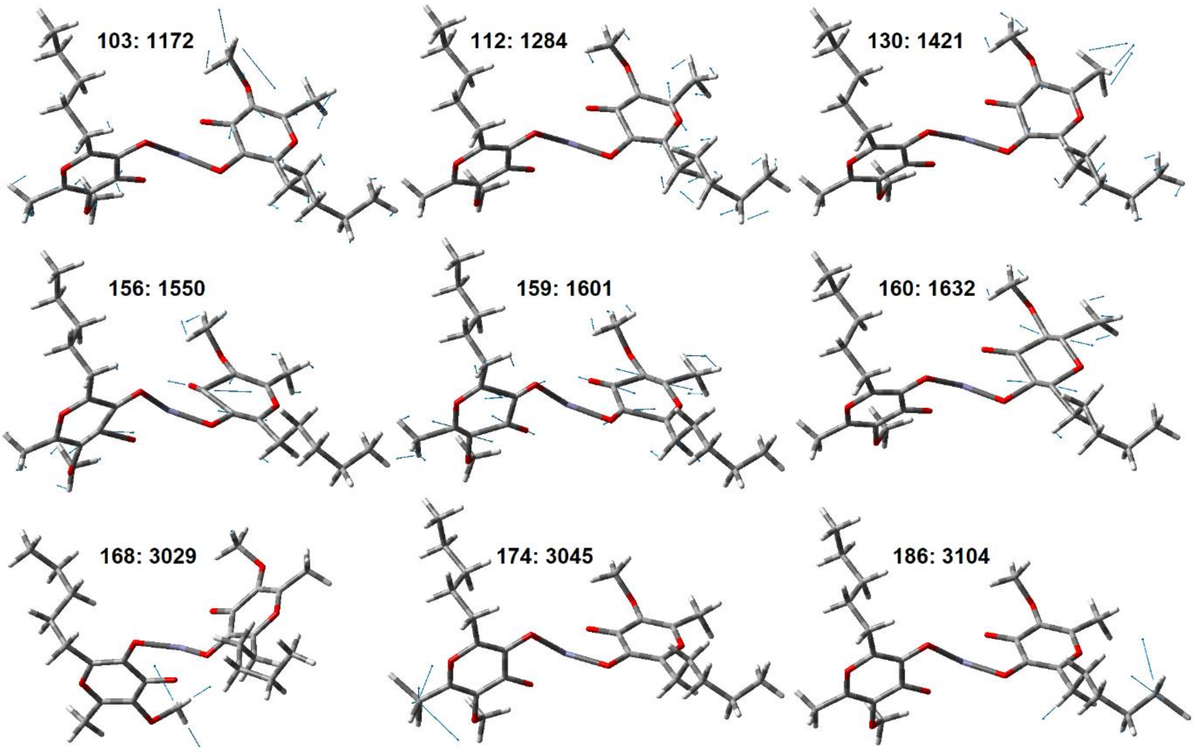

Figure S27. Images of displacements for selected normal modes as computed for Zn(alx)$_2$ under tetrahedral geometry. For each normal mode the numbering used in the Fig. S26 is reported together with the corresponding frequency in cm^{-1}.

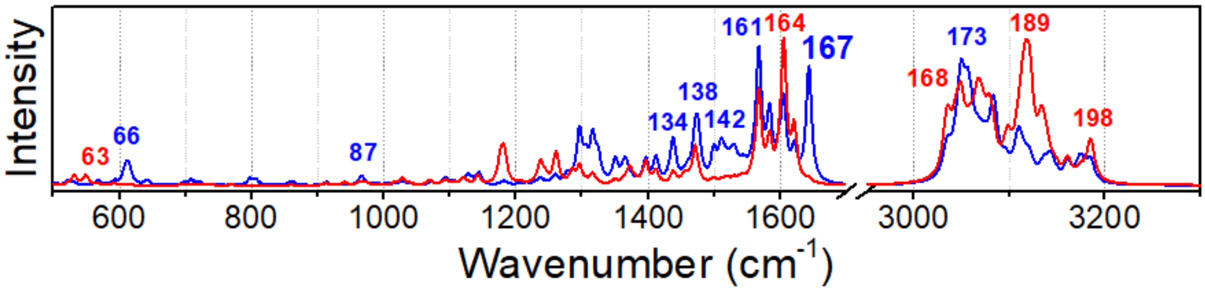

Figure S28. Infrared (red line) and Raman (blue line) spectra computed for Zn(alx)$_2$, when associated with PTEN. Modes' numbering account additional 3 translations and 3 rotations of the complex in respect to protein cavity: they are the lowest frequency modes. Intensities of spectra in the middle and high frequency ranges are scaled for comparative visibility. Raman spectrum is computed accounting excitation wavelength at 532 nm.

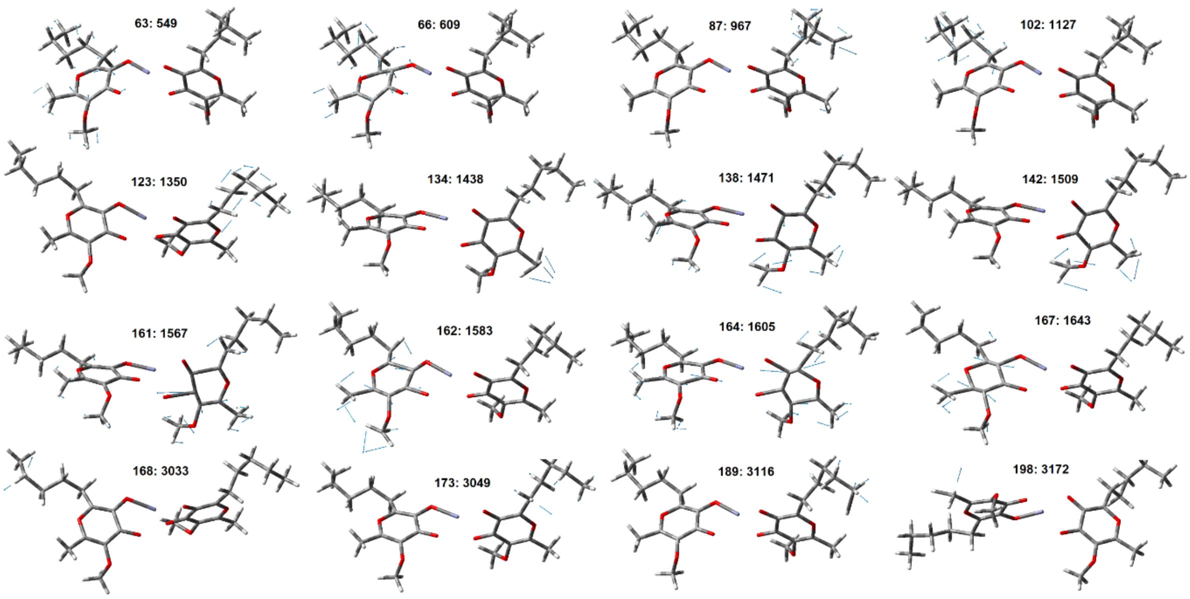

Figure S29. Images of displacements for selected normal modes as computed for Zn(alx)$_2$, when associated with PTEN. For each normal mode the numbering used in the Fig. S28 is reported together with the corresponding frequency in cm^{-1}.

For the Zn(alx)$_2$ complex embedded in the protein environment, the normal modes of the dominant IR and Raman bands shift to the blue. A nearly degenerated doublet of IR transitions 106 and 107 to involve CH bendings of the methoxy and methyl group peak at 1181 cm^{-1}. Mode 161 (at 1567 cm^{-1}) accounts C_2-C_3&C_4-O_4/C_4-C_5 admixed with CH bendings on the same ligand. The infrared dominant peak at 1605 cm^{-1} is due to the mode 164 to demonstrate (C_2-C_3/C_4-O_4&C_5-C_6)/(C'_4-O'_4/C'_5-C_6) delocalized over both ligands to admix with their CH bendings. The dominant Raman peak at 1593 cm^{-1} accounts a nearly degenerated doublet of C_2-C_3&C_5-C_6 and C'_2-C'_3/C'_5-C'_6 vibrations localized on each of the ligands. Overall, the computed vibrations of the complex in PTEN demonstrate wider spectral distributions of the resonances and stronger localization tendencies reflecting lower symmetry due to structural distortions resulting from the hindered interactions with the protein.

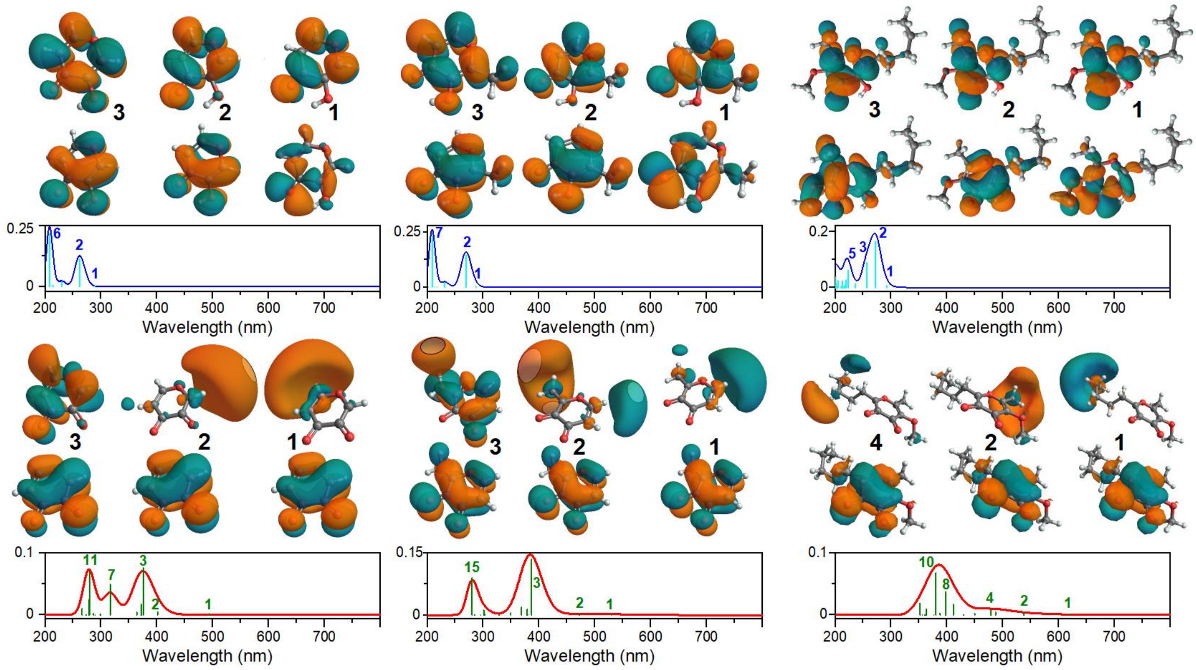

Figure S30. Images TDDFT computed NTO transitions and optical absorption spectra of 3-hydroxy-4-pyrone, maltol and allixin, when deprotonated (upper set) and protonated (lower set).

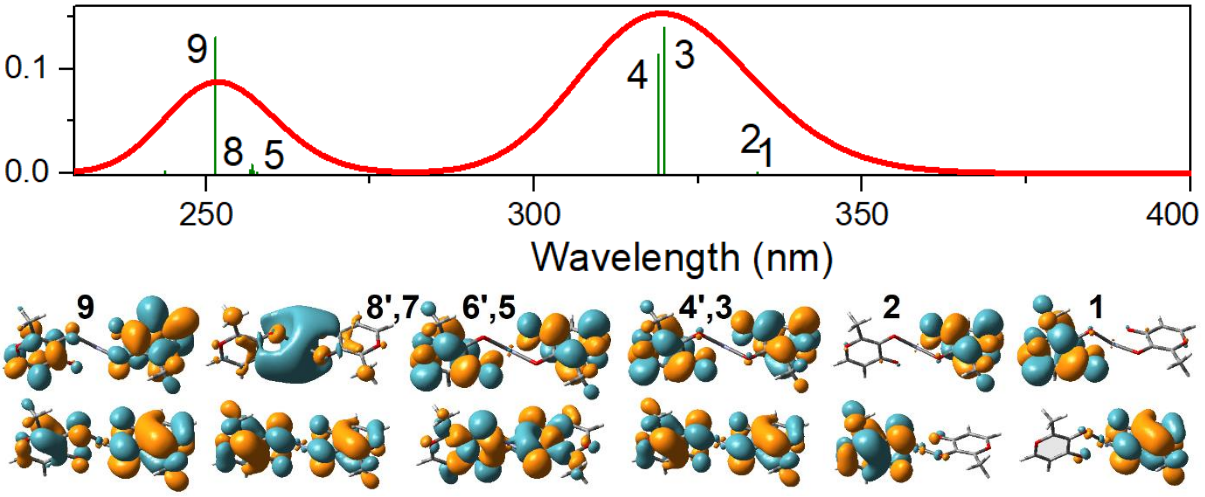

Figure S31. TDDFT computed NTO transitions and optical absorption spectra for Zn(mal)$_2$ under tetrahedral geometry. Transitions 3 and 4 are similar in nature: prime sign indicates opposite phase of the ligand orbital components in the lower (hole) state. Transitions 5 and 6 are similar in nature: prime sign indicates opposite phase of the ligand orbital components in the upper (electron) state. Transitions 7 and 8 are similar in nature: prime sign indicates opposite phase of the ligand orbital components in the lower (hole) state.

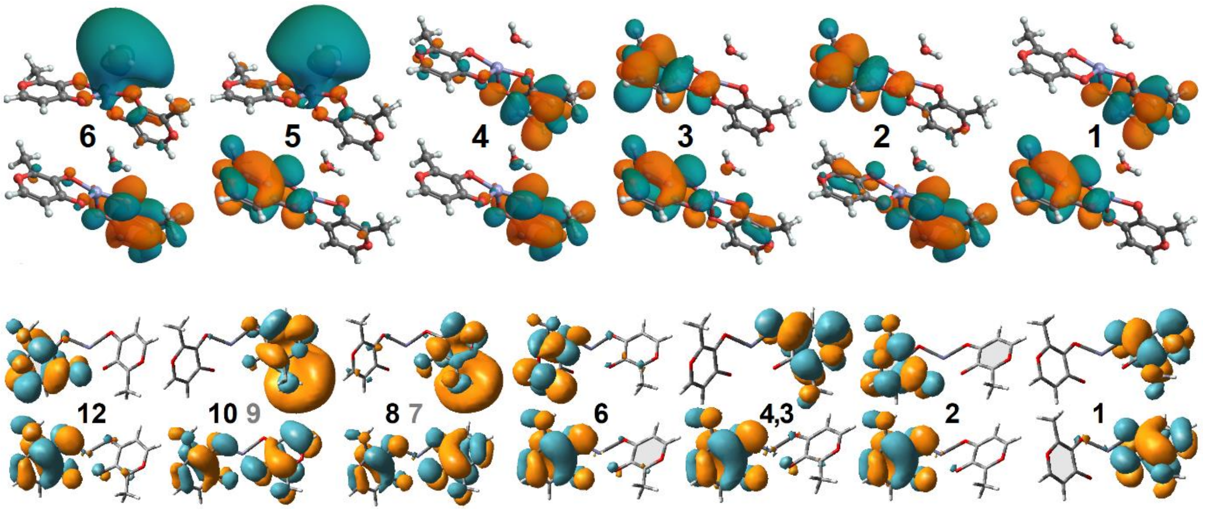

Figure S32. NTO pairs for selected resonance (as numbered) for $Zn(mal)_2{\cdot}H_2O$ optimized in vacuum (top) and for $Zn(mal)_2$ when associated with PTEN protein (bottom). The corresponding optical electronic spectral properties are shown in Fig. 8A in the main text. Transitions 7 and 8 (as well as 9 and 10) are similar in nature: black and grey colors indicate opposite phase of the ligand orbital components in the lower (hole) state.

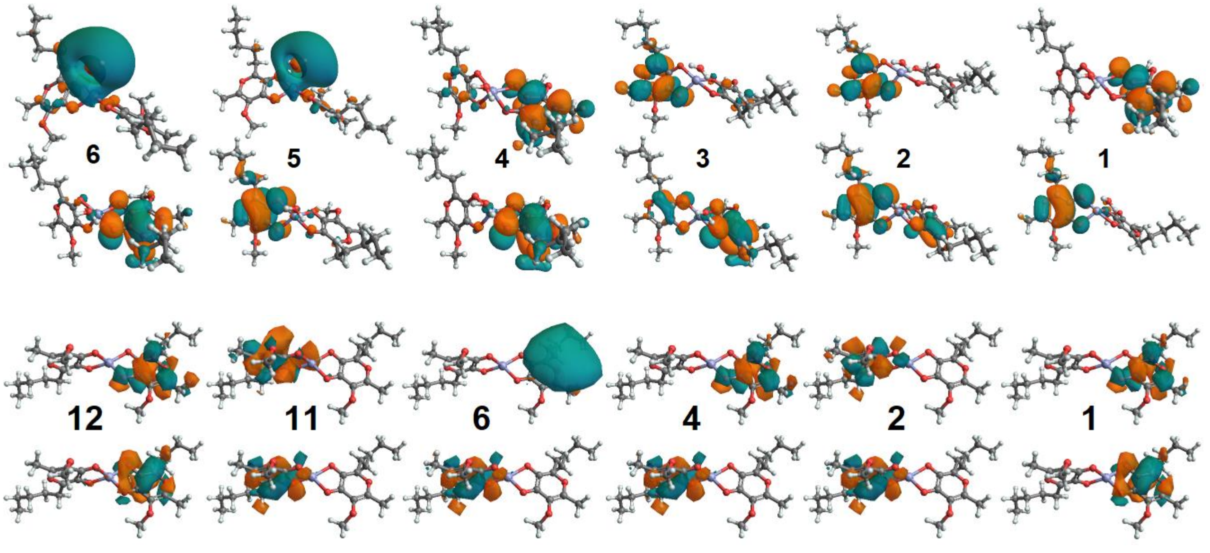

Figure S33. NTO pairs for selected resonance (as numbered) for $Zn(alx)_2{\cdot}H_2O$ optimized in vacuum (top) and for $Zn(alx)_2$, when associated with PTEN protein (bottom). The corresponding optical electronic spectral properties are shown in Fig. 8C in the main text.

Conditioning of Neural Net to predict d-d resonances according to Coulomb interactions between atoms of a complex and proximal atoms of embedding protein.

First, we introduce a geometry criterion to collect coordinates of protein atoms, which are next to the reaction centre, where a complex would bind. Specifically, using a pdb file, we may select suitable N-H coordinates and define axis of the helix, which points to the reaction centre. Then, we extend the axis further into the reactive space (red arrow in the Fig. S34), and set up a radius of a cylindrical space to collect atoms within: see green spheres in the Fig. S34 and details in the Mathematica notebook files, namely 1_ReactiveSpace_PTP1B_*_Coulomb_Matrix.nb.

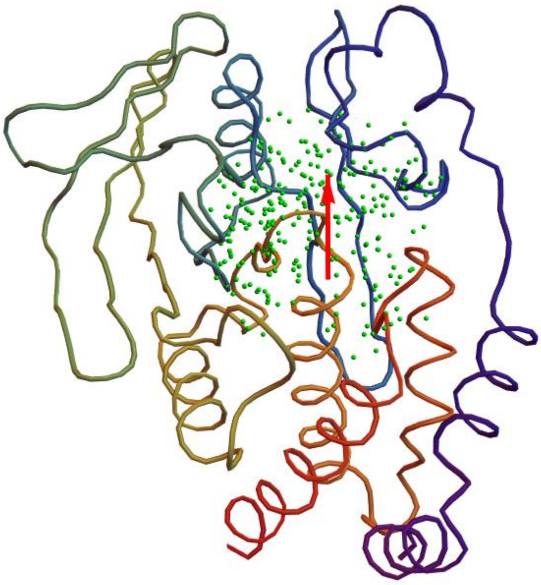

Figure S34. Collected atoms (green spheres), which are next to the reaction centre of PTP1B protein according to 1onz.pdb and geometric criteria as described in 1_ReactiveSpace_PTP1B_*_Coulomb Matrix.nb files.

Setting own libraries for charges and type of atoms, and using DFT computed coordinates of a complex in a protein, we compute Coulomb forces for each atomic pair. The numbers may be stored in a Coulomb Matrix (CM) with dimensions *m* x *n*, where *m* is the number of the selected protein atoms, and *n* is the number of complex atoms. The matrix is not square. Next, taking CM.Transpose[CM] vector product, we may regularize the Coulomb matrix preparing its squared presentation of *m* x *m* dimensions, according to the number of selected protein atoms. This task is accomplished by the notebook Mathematica files
1_ReactiveSpace_PTP1B_hp_Coulomb_Matrix.nb
1_ReactiveSpace_PTP1B_alx_Coulomb_Matrix.nb
for the exemplary cases of the $VO(3hp)_2$ and $VO(alx)_2$ complexes in the PTP1B protein after QM/MM optimization.

In result of DFT QM/MM studies, we have lists of energies (wavelengths) of four d-d transitions for:
a) $VO(3hp)_2$ in vacuum;
b) $VO(alx)_2$ in vacuum;
c) $VO(3hp)_2$ in PTPB;
d) $VO(3alx)_2$ in PTP1B.

In the cases (a) and (b), since the complexes are in vacuum, Coulomb matrices reflecting interactions with the protein are zero, CM0.

In Mathematica notebook 2_Matrix_Vector_Training.nb, we address AI assisted regression of the wavelengths of four d-d resonances on Coulomb matrices for the complexes in vacuum (CM0) and in the PTP1B environment (CM_{vhpP} and CM_{valxP}): we relate values of 4 wavelengths on the 3 matrices.

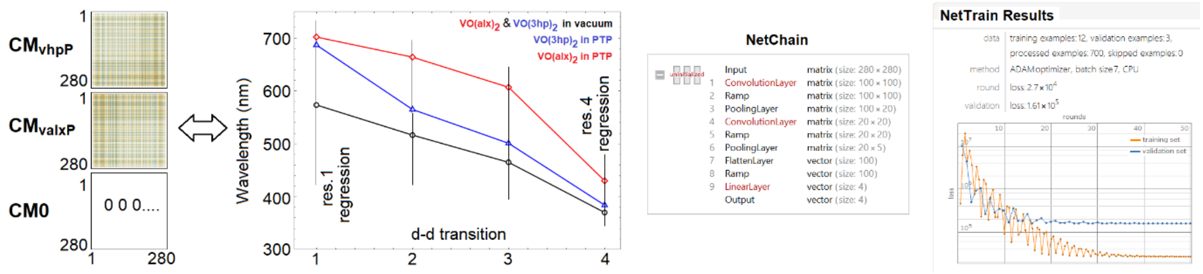

Figure S35. Assignments and Net training outline as programmed in 2_Matrix_Vector_Training.nb file.

To train an effective Neural Net, we prepare Coulomb matrix mixtures to associate with the corresponding wavelength vectors mixtures, for examples:

$0.1*CM0 + 0.4* CM_{vhpP} \rightarrow 0.1*\{w1_0,w2_0,w3_0,w4_0\} + 0.4*\{w1_{vhpP},w2_{vhpP},w3_{vhpP},w4_{vhpP}\}$

or

$0.3*CM_{vhpP} + 0.2* CM_{valxP} \rightarrow 0.3*\{w1_{vhpP},w2_{vhpP},w3_{vhpP},w4_{vhpP}\} + 0.2*\{w1_{valxP},w2_{valxP},w3_{valxP},w4_{valxP}\}$.

Here,

$\{w1_0,w2_0,w3_0,w4_0\}$ is the vector of the wavelengths of the 4 d-d transitions as computed for a complex in vacuum;

$\{w1_{vhpP},w2_{vhpP},w3_{vhpP},w4_{vhpP}\}$ is the vector of the wavelengths of the 4 d-d transitions computed for $VO(3hp)_2$ in PTP1B;

$\{w1_{valxP},w2_{valxP},w3_{valxP},w4_{valxP}\}$ is the vector of the wavelengths of the 4 d-d transitions computed for $VO(3alx)_2$ in PTP1B.

Mathematica notebook 2_Matrix_Vector_Training.nb provides a primer for prediction of wavelengths of d-d resonances according to Coulomb matrices computed according to the results of our QM/MM calculations. The approach may suggest expanding the initiative to account other systems to vary complexes, or edit or vary the protein.
